# The bone lid technique in lateral sinus lift: a systematic review and meta-analysis

**DOI:** 10.1186/s40729-022-00433-3

**Published:** 2022-08-29

**Authors:** Lucia Schiavon, Alessandro Perini, Giulia Brunello, Giada Ferrante, Massimo Del Fabbro, Daniele Botticelli, Fouad Khoury, Stefano Sivolella

**Affiliations:** 1 Department of Neurosciences, Dentistry Section, University of Padova, Via Giustiniani 2, 35128 Padua, Italy; 2 Department of Oral Surgery, University Clinic of Düsseldorf, Moorenstrasse 5, 40225 Düsseldorf, Germany; 3 Department of Biomedical, Surgical and Dental Sciences, Università Degli Studi di Milano, Via Commenda 10, 20122 Milan, Italy; 4 Fondazione IRCCS Ca’ Granda Ospedale Maggiore Policlinico, Via Francesco Sforza, 35, 20122 Milan, Italy; 5 ARDEC Academy, Viale Pascoli 67, Rimini, Italy; 6 Department of Oral and Maxillofacial Surgery, University of Munster, Waldeyerstr. 30, 48149 Munster, Germany; 7 International Dental Implant Center, Private Clinic Schloss Schellenstein, Am Schellenstein 1, 59939 Olsberg, Germany

**Keywords:** Sinus floor augmentation, Maxillary sinus, Bone regeneration

## Abstract

**Objective:**

This systematic review aimed at assessing the effect of the repositioned bone lid on bone augmentation in lateral sinus lift in pre-clinical in vivo and clinical studies. Secondary aims were to report on the healing of the bone window and to assess the implant survival rate.

**Material and methods:**

Animal and human studies comparing lateral maxillary sinus floor elevation in combination or not with the repositioned bone lid were retrieved from MEDLINE (PubMed), Web of Science and Cochrane online library. Studies published in English up to April 2022 and reporting on histological and/or radiographic outcomes were considered. Case reports, case series and reviews were excluded. A hand search was also conducted. Risk of bias was assessed and meta-analysis performed to investigate the effect of the bone lid on new bone formation.

**Results:**

After screening, 5 animal studies (4 in rabbits, 1 in sheep) and 2 clinical studies (1 RCT, 1 case–control) were included. Meta-analysis confirmed a higher new bone formation in rabbits at 2 and 8 weeks using the bone lid. The two clinical studies investigated lateral sinus lift with concomitant implant placement and reported similar results and high short-term implant success rate in both test and control groups.

**Conclusions:**

The meta-analysis provided moderate evidence that the repositioned bone lid favored the formation of new bone to a higher extent as compared to resorbable membranes in animal studies. Implant success seems not to be influenced by the technique in the short term.

## Introduction

Maxillary sinus floor elevation is a common surgical procedure used to increase the bone volume in the atrophic posterior maxilla prior to dental implant placement. It can be achieved through a lateral approach, with simultaneous or delayed implant insertion [1]. The lateral sinus lift procedure is indicated in cases of severe bone resorption, not allowing standard implant placement nor implant insertion in combination with a crestal approach [2, 3]. This involves the fashioning of a bone window in the lateral wall of the maxillary sinus and the elevation of the Schneiderian membrane [4]. The resulting void space can be filled with autologous bone [5], bone substitutes [6], a combination of both [7], or blood clot [8]. The lateral bone window can be scraped, can be left intact and rotated inwards attached to the membrane or, it can be removed and then either discarded, ground for obtaining bone chips, or repositioned back in place [9].

At the end of the surgery, the antrostomy can be closed suturing directly the muco-periosteal flap over the grafting material, or it can be covered with a resorbable [3] or non-resorbable membrane [10]. The use of a membrane seems to positively influence the healing outcomes, preventing graft migration, reducing soft tissue ingrowth and enhancing new bone formation [3, 11, 12].

As an alternative to membranes, the bone lid technique has been proposed [13]. This approach consists in fashioning and removing a bone lid or window, which is replaced into its original position at the end of the surgery. This should act as an autogenous barrier with osteoconductive properties, thus further accelerating new bone formation and enhancing graft integration [14–19]. The use of thin bone cutting instruments and a beveled osteotomy design facilitate the exact repositioning of the lid and its revascularization [14]. The lid could be stabilized with additional fixation devices, such as mini-plates and mini-screws, if needed. In a recent scoping review, a correlation between the fixation method and the risk of bone lid resorption and necrosis could not be determined [20]. Independently of the fixation, the reported rate of these complications was approximately 2.5% [20].

The reposition of the bone window has been described for several indications in oral surgery with favorable outcomes [14, 21–28], including its application for sinus floor elevation [29–35].

The amount of new bone formation is generally considered the most appropriate parameter to determine the success of the lateral sinus lift [36]. Therefore, the primary aim of the present systematic review and meta-analysis was to evaluate the effect of the repositioned bone lid on bone augmentation in lateral sinus lift in pre-clinical in vivo and clinical studies in terms of histological and radiographic outcomes. Secondary aims were to report on bone window healing and to assess the implant survival rate.

## Materials and methods

The present systematic review and meta-analysis was conducted in accordance with the Preferred Reporting Items for Systematic Reviews and Meta-Analyses (PRISMA) guidelines [37].

The protocol for this review was registered with the international prospective register of systematic reviews (PROSPERO) with registration n. CRD42020184317.

### Focal question

The focused “PICOS” (population, intervention, comparison, outcome, study) question addressed was the following: “Is there any difference in terms of new bone formation after lateral sinus lift in combination or not with the repositioned bone lid in animal and human studies?”.

P: animal and human maxillary sinus.

I: lateral sinus lift in combination with bone lid technique.

C: lateral sinus lift without the use of bone lid technique.

O: new bone formation.

S: animal and human controlled studies.

### Search strategy and eligibility

Animal and human studies comparing the lateral maxillary sinus floor elevation in combination or not with the repositioned bone lid were searched in the MEDLINE online library via PubMed, Web of Science and the Cochrane Central register of Controlled Trials (The Cochrane Library), up to 5^th^ April 2022. Search strategies are reported in Table 1. Search terms were used alone or in combination using Boolean operators OR, AND. Only animal and clinical studies published in English language were considered. Narrative and systematic reviews, single case reports, case series or technical reports were not considered.

**Table 1:** Details of search strategies

Database	Search strategies
MEDLINE via PubMed	("bone lid"[All Fields] OR "bony lid"[All Fields] OR "bone window"[All Fields] OR "bony window"[All Fields]) AND ("maxillary sinus"[All Fields] OR "lateral sinus lift"[All Fields] OR "sinus floor augmentation"[All Fields] OR "sinus floor elevation"[All Fields] OR "sinus lifting"[All Fields] OR "sinus lift"[All Fields])
Web of Science	("bone lid" OR "bony lid" OR "bone window" OR "bony window") AND ("maxillary sinus" OR "lateral sinus lift" OR "sinus floor augmentation" OR "sinus floor elevation" OR "sinus lifting" OR "sinus lift") (All Fields)
Cochrane	(“bone lid” OR “bony lid” OR “bone window” OR “bony window”) AND (“maxillary sinus” OR “lateral sinus lift” OR “sinus floor augmentation” OR “sinus floor elevation” OR “sinus lifting” OR “sinus lift”) in Title Abstract Keyword

The search was complemented by hand-searching on the major journals of the field of oral and maxillofacial surgery and implant dentistry. In addition, a hand search was performed through the reference list of the included studies.

Only animal and human studies reporting on histological and/or radiographic outcomes were selected. Included studies had to compare the repositioned bone lid (test) versus other approaches (control) for lateral sinus lift. To be eligible, they had to provide details on the sample size, the surgical procedure, the grafted material (if utilized). They also had to clearly define the outcomes used to evaluate the success or failure of the treatment in terms of new bone formation within the elevated space. Data on bone lid healing and the survival rate of dental implants, if positioned, were also recorded.

### Selection of the studies

Study selection was carried out by two independent researchers (GB and LS) using a two-stage screening procedure. In the first phase, only titles and abstracts of the retrieved articles were evaluated. Subsequently, full texts of the eligible articles were screened to check if they met all inclusion criteria. For articles excluded after full-text evaluation, the main reason for exclusion was reported. Disagreements between the reviewers were solved by discussion, and eventually by consulting a third reviewer (MDF). For both steps, the inter-reviewer agreement was assessed by means of the Cohen’s Kappa coefficient.

### Data extraction

Data were extracted separately by two reviewers (GF and LS), for animal and clinical studies, respectively. Qualitative data extracted from the included studies were synthesized in analytic tables.

The main variables extracted from each included animal study were the following: animal model; sample size; biomaterial(s) used in the test and control side (if any); time of killing; bone cutting instruments; fixation method; any outcome variable used to evaluate treatment success; main findings.

For human studies the following variables were recorded and summarized: study design; sample size, biomaterial(s) utilized (if any); follow-up duration; bone cutting instruments; fixation method; number of implants positioned and timing of insertion (if applicable); any outcome variable used to evaluate treatment success; main findings.

### Quality assessment and risk-of-bias analysis

For pre-clinical in vivo studies, the quality of the studies was assessed independently by two reviewers (GF and AP) using the updated ARRIVE (Animals in Research: Reporting In Vivo Experiments) guidelines [38], which evaluates 21 items. The risk-of bias of the animal studies was assessed by using the SYRCLE tool [39], structured in 10 items.

For clinical studies, risk–of-bias assessment was conducted independently by two reviewers (GB and LS). The revised Cochrane risk-of-bias tool for randomized trials (RoB 2) [40], structured in five bias domains, was used. For observational clinical studies the risk of bias was assessed with the ROBINS-I tool (Risk Of Bias In Non-randomized Studies of Interventions) [41].

Any disagreement was resolved by discussion and, if needed, a third reviewer (MDF) was contacted.

### Statistical analysis

Descriptive statistics was done of the included studies by summarizing the total number of animal/patients and cases treated with and without the bone lid technique. If at least three homogenous studies (in terms of species, follow-up time and outcome variables) comparing cases treated with and without bone lid were found, a meta-analysis was undertaken. The estimates of the effects of using the bone lid technique were expressed as odds ratio (OR) for dichotomic outcomes or standardized mean difference (SMD) for continuous variables, as appropriate, together with 95% confidence intervals. ORs or SMDs were combined using a fixed-effects model (Mantel–Haenszel method) or a random-effects model, according to heterogeneity. Heterogeneity among studies was assessed by using the *Q* Cochrane test, and *I*^2^. Fixed-effects meta-analysis was used when the heterogeneity was small (*i*^2^ < 60%, *P* > 0.05), otherwise a random-effects model analysis was undertaken. The statistical evaluation was conducted considering the patient/animal as the analysis unit. *P* = 0.05 was considered as the significance level.

## Results

**Fig. 1:**
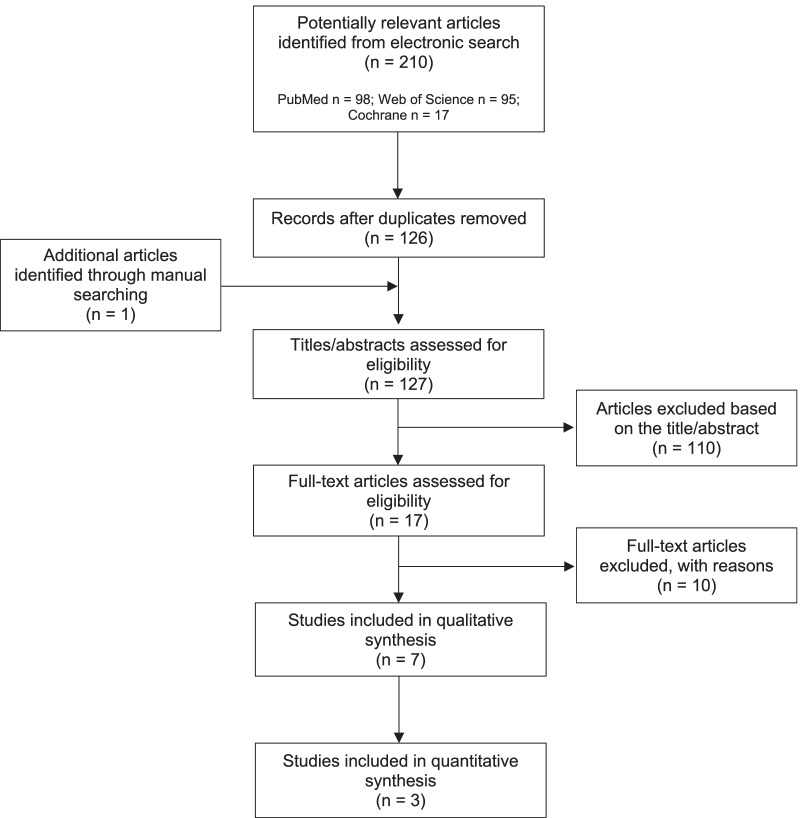
Flow-chart

The flow-chart of the selection process is illustrated in Fig. 1. The electronic search yielded a total of 126 articles after the removal of duplicates. One additional article was found by hand-searching. After the first step, 17 articles were selected (inter-reviewer agreement κ = 0.94). The evaluation of the full texts led to the inclusion of 5 pre-clinical in vivo [17–19, 29, 31] and 2 clinical studies [32, 42] (inter-reviewer agreement κ = 1). At this second phase of screening, the majority of the articles were excluded due to the use of other methods rather than the bone lid technique [11, 43–47]. Other reasons for exclusion were the use of the bone lid technique in both test and control groups [48, 49], the absence of a control group [50], and the use of the bone lid technique for other clinical indications [14]. Three in vivo animal studies were considered eligible for quantitative evaluation [17, 18, 29].

### Pre-clinical animal studies

In the 5 included animal studies, rabbits were used in four [17–19, 29], and one study was conducted in sheep [31]. The characteristics and the main results of the studies are presented in Table 2. All the studies used split-mouth animal models. Bone window was repositioned at the test site, while at the control site, the antrostomy was covered with a collagen [17–19, 29] or a polylactic resorbable membrane [31]. In none of the included studies dental implants were placed.

**Table 2:** Summary of the characteristics and main results of pre-clinical animal studies

Refs.	Animal model	Sample size (no. animals)	Test side- bone lid biomaterial(s)	Control side Biomaterial(s)	Killing (weeks)	Bone cutting instruments	Bone lid fixation	Assessment method(s)	Main findings
Perini et al. [31]	Sheep	8	β-TCP	β-TCP + polylactic membrane	16	Round bur	Cyanoacrylate-based surgical glue	Histological analysis Histomorphometric analysis	Histological analysis: new bone surrounding and partially interpenetrating the biomaterial’s particles, in continuity with the lateral and medial walls of the sinus and with the repositioned bone plate. The bone window appeared connected by new formed bone with the edge of the antrostomy and the center of the elevated area. No remnants of cyanoacrylate Histomorphometric analysis: higher bone regeneration and bone interpenetration into the graft in the close-to-window region in test than in control group (bone regeneration: 22.1 ± 12.6%; vs 7.5 ± 4.5%; *P* = 0.028 and bone interpenetrated: 66.1 ± 14.7% vs 44.2 ± 15.1%; *P* = 0.046, respectively). Higher amount of soft tissue in the middle of the elevated space in the control than in the test site (37.5 ± 10.1% vs 29.7 ± 4.9%; *P* = 0.046) In other areas, no differences between the two groups in terms of new bone % and graft %. More bone regenerated at the edges of the antrostomy in the test sites compared with the control ones, not statistically significant Complications: loss of biomaterial in one sinus of the test group (associated with sinusitis) and one sinus of the control group; loss of one repositioned bone lid (probably during histological processing)
Omori et al. [29]	Rabbit	18	Collagened cortico-cancellous porcine bone	Collagened cortico-cancellous porcine bone + collagen membrane	2,4,8	Sonic instrument	Cyanoacrylate-based surgical glue	Histological analysis Histomorphometric analysis	Histological analysis: at 2 weeks, small amounts of new bone, originating from the bony sinus walls, in both groups. New bone density increased proportionally to the healing period, both in test and control group. Small residual defects around the margin of the repositioned bone plate in the test sites, and in the center of the antrostomy in the control ones. Surgical glue remnants still present after 8 weeks. Xenograft resorption increased with the healing period Histomorphometric analysis: *2 weeks*: both in test and control groups, small amount of new bone (2.5 ± 2.4% vs 1.6 ± 1.0%, *P* < 0.05), predominantly proximal to the bony walls (4.0 ± 3.8% vs 3.1 ± 2.3%). No bone adjacent to the sinus mucosa and in the middle area. New bone % at the edge of the antrostomy were 6.4 ± 12.5% (test) and 3.2 ± 1.7% (control). No bone regenerated in the central region of the control site *4 weeks*: new bone increased to 7.9 ± 6.5% and 8.5 ± 9.7% (*P* < 0.05), respectively, in the test and control site with highest amounts near the bone walls (11.0 ± 9.5% vs 10.0 ± 9.5%). At the edge of the antrostomy new bone % increased both in test and control groups (24.7 ± 16.8% vs 18.1% ± 11.6%) *8 weeks:* new bone values were 22.7 ± 13.6% and 23.9 ± 14.4% (*P* < 0.05), respectively, at the test and control site with lower amount in the sub-mucosa region. New bone at the edge of the antrostomy: 33.9 ± 19.3% (test) and 37.4 ± 19.2% (control) Complications: 3 sinus membrane perforation in the control group repaired with collagen membranes (two at 2 weeks and one at 4 weeks, still visible at the histological analysis). Loss of biomaterial in one sinus of the 2 weeks control group
Moon et al. [17]	Rabbit	16	β-TCP	β-TCP + collagen membrane	1, 2, 4, 8	Piezosurgery (saw)	None	Histological analysis Histomorphometric analysis	Histological analysis: new bone thickness and density as well as new bone around β-TCP particles increased following the healing periods. Higher and faster bone regeneration in the test sites. No signs of inflammation. TRAP staining revealed an increasing number or osteoclast from 1 to 4 weeks, which decreased at 8 weeks in all groups Histomorphometric analysis: *control group:* new bone % increased at 1, 2,4 and 8 weeks (0.6 ± 0.10%; 6.35 ± 1.25%; 13.02 ± 1.76% and 21.74 ± 2.40%) with significant difference between each timepoint and the previous one (*P* < 0.05). *Test group:* new bone significantly increased between each timepoint and the former (0.58 ± 0.21%; 11.15 ± 1.48%; 19.81 ± 2.22% and 31.28 ± 3.51%, respectively, at 1, 2, 4, and 8 weeks) (*P* < 0.05). Lamellar bone/ new woven bone ratio significantly higher in the test group at 4 and 8 weeks. β-TCP resorption significantly higher in test group at 8 weeks Complications: None
Sohn et al. [19]	Rabbit	20	None	DBBM + collagen membrane	1, 2, 4, 6, 8	Piezosurgery (saw)	Mini-screws	Histological analysis Immunohistochemical analysis	Histological analysis: *Test group*: new woven bone along the repositioned bone window at 1 week. New bone expanding from the repositioned bone window to the center of the elevated area at 2 weeks. At 4,6 and 8 weeks, new bone thickness, maturation, and density progressively increased *Control group*: no new bone around the biomaterial at 1 week. At 2 weeks new bone mainly along the elevated sinus mucosa. At 4 weeks, presence of osteocytes and new bone bound to the biomaterial particles. At 6 weeks, presence of more mature and abundant new bone and particles surrounded by dense and mature new bone Immunohistochemical analysis: *test group*: positive cells for PCNA presence along the floor of the replaceable bone window and the elevated sinus membrane from 1 to 4 weeks. Weak expression of positive cells for PCNA on the new bone surface. Strong expression of type I collagen in new bone on the floor of the repositioned bone window and weak expression of type I collagen in the surrounding soft tissue. Osteocalcin expression mainly found on the new bone along the floor of the repositioned bone window after 1 week. *Control group:* few PCNA-positive cells found at the new bone surface under the elevated sinus membrane. At 2 weeks, more PCNA-positive cells were observed in the biomaterial particles and presence of osteoblasts on the new bone surface. Weaker expression of PCNA-positive cells at 4, 6 and 8 weeks. Strong expression of type I collagen on osteoblasts and new bone surface after 2 weeks. Osteocalcin was observed on the surface of new bone around the biomaterial particles, but not along the collagen membrane at 1 week Complications: none
Sohn et al. [18]	Rabbit	20	None	DBBM + collagen membrane	1, 2, 4, 6, 8	Piezosurgery (saw)	Mini-screws	Histological analysis Histomorphometric analysis	Histological analysis: *Test group:* initial new bone formation under the elevated sinus membrane and the floor of the replaced bone window after 1 week. Active bone regeneration with presence of osteoblasts and osteoclasts at 4 weeks. New bone thickness and density constantly increased, up to 8 weeks. *Control group:* no new bone around the biomaterial nor under the collagen membrane at 1 week. Bone regeneration started from the 2^nd^ week and constantly increased till the 8^th^ week. Higher and faster bone regeneration along the elevated sinus membrane than around the graft TRAP staining: in both test and control group, osteoclasts number increased from 1 to 4 weeks, and decreased at 6 and 8 weeks, compared with the 4 weeks timepoint. No statistically significant differences Histomorphometric analysis: *test group:* bone regeneration 1.52 ± 0.79%; 11.33 ± 2.28%; 43.09 ± 5.23%; 55.62 ± 4.81% and 65.96 ± 2.99%, respectively, at 1, 2, 4, 6 and 8 weeks, with significant difference between each timepoint (P < 0.001). Control group: newly formed bone: 0.55 ± 0.29%; 3.54 ± 1.02%; 14.36 ± 2.71%; 21.94 ± 1.89% and 23.26 ± 2.07% respectively at 1, 2, 4, 6 and 8 weeks, with no significant differences only between 6 and 8 weeks. Significantly faster regeneration and denser new bone in the test group compared with the control one Complications: None

*β-TCP* β-tricalcium phosphate, *DBBM* deproteinized bovine-derived bone mineral, *PCNA* proliferating cell nuclear antigen

In three studies the same biomaterial was placed both in test and control groups, i.e., β-tricalcium phosphate (β-TCP) [17, 31] and collagenated cortico-cancellous porcine bone [29]. In the remaining two studies, repositioned bone lid combined with maxillary sinus lift without bone grafting materials was compared to the use of deproteinized bovine bone mineral (DBBM) with a resorbable membrane [18, 19].

When rabbits were used as animal models, euthanasia was performed at multiple time points in a range between 1 and 8 weeks, while a single time point was set for sheep, at 4 months after surgery. Reported cutting devices for the antrostomy were piezosurgery [17–19], round burs [31] or sonic device [29]. At the end of sinus augmentation, the bone window was fixed with cyanoacrylate-based surgical glue [29, 31] or mini-screws [18, 19]. In Moon et al., no fixation method was reported [17]. In all the included studies, bone healing was assessed through histological analysis and details are provided in Table 2. In four studies histomophometric evaluation was performed [17, 18, 29, 31], and in one immunohistochemical analysis was conducted [19]. New bone was found mainly originating from the sinus bone walls. In rabbit models, where euthanasia was performed at multiple times, new bone density and thickness were found to increase overtime.

In the repositioned bone window sites, at the histological analysis the bone lid appeared well integrated with the margin of the antrostomy, while residual defects were found when a membrane had been placed over the antrostomy [17, 29, 31].

**Fig. 2:**
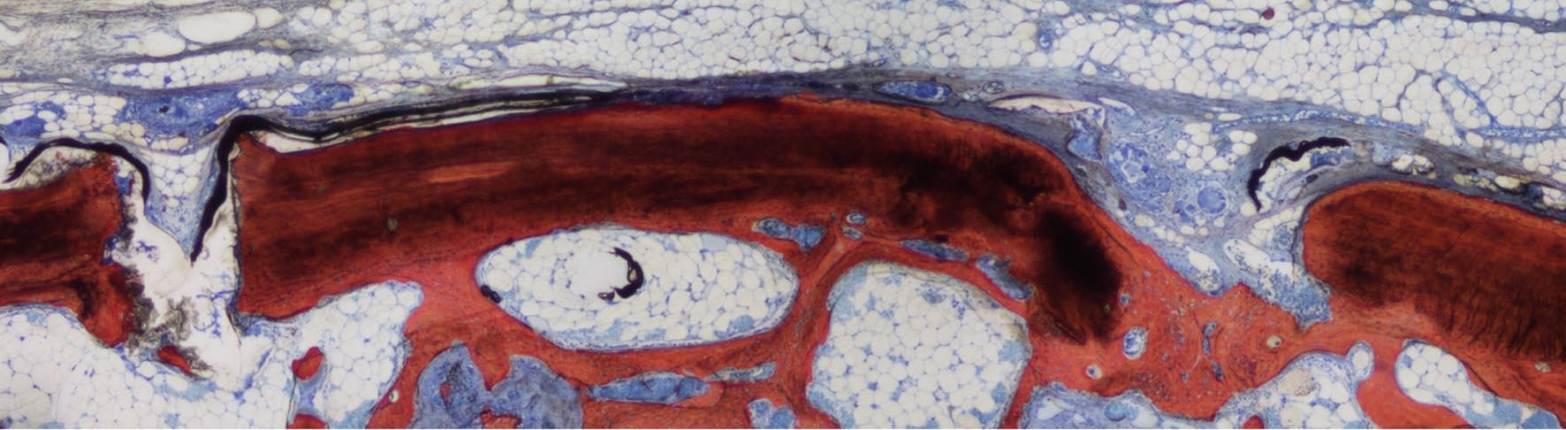
Ground section representing the healing after 8 weeks. New bone continued to increase in proportion both at test and control (not shown) site in the antrostomy region. Original magnification × 100. Stevenel´s blue and alizarin red stain. From Omori et al. [29]

In the study in rabbits in which the cyanoacrylate was used as a fixation method [29], residual glue at the interface with the repositioned bone window interfered with bone lid healing and integration (Fig. 2). In the study in sheep no cyanoacrylate remnants were observed after 4 months [31]. The difference in glue presence and influence on healing might be explained by the difference in dimensions of the two bone windows so that, in the sheep study, the glue resulted to be far away from the plane of the histological slide.

In the three papers [17, 29, 31] where the same biomaterial was used in both groups and the repositioned bone lid was compared with a resorbable membrane to close the antrostomy, a higher bone regeneration was found in the bone lid groups, which was reported to be statistically significant in one study [17]. In the experimental study on sheep (Fig. 2), only in the close-to-window area significant differences were observed between the two groups in terms of new bone and bone interpenetrated to the graft [31]. Comparing the repositioning of the bone window without bone grafting vs DBBM covered by a collagen membrane, faster and higher new bone formation was observed in the bone lid sites [18]. Indeed, histomorphometric analysis confirmed a significantly higher percentage of newly formed bone, calculated as a ratio of newly formed bone area to the total augmented area except for the DBBM particle area in the control group, in the bone lid group as compared to the control one from 2 to 8 weeks [18].

### Clinical studies

**Fig. 3:**
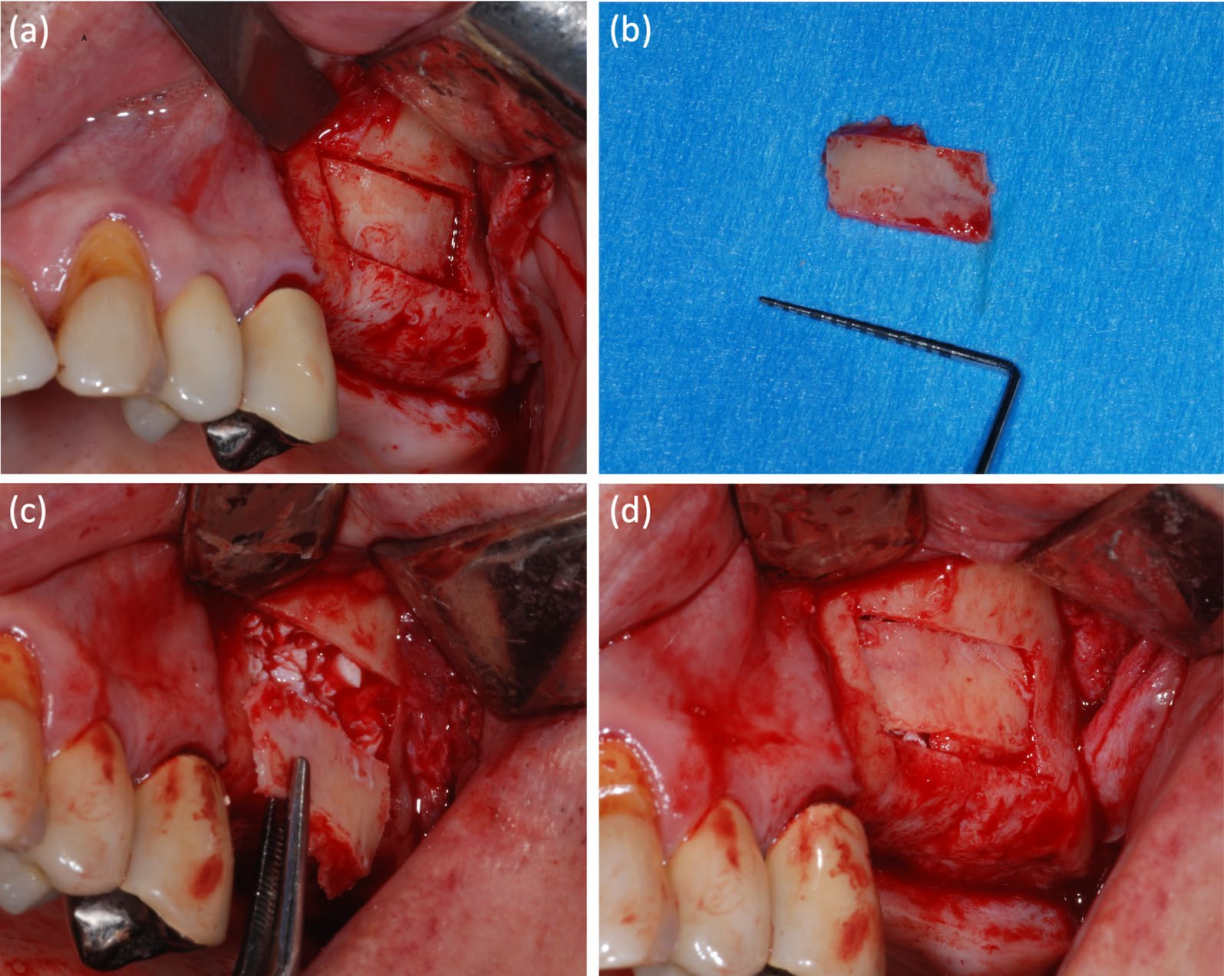
**a**–**d** Photo sequence of a lateral sinus lift surgery with repositioned bone lid and heterologous bone graft

One randomized controlled trial (RCT) [42] and 1 case–control study [32] were included, accounting for 15 bone lids. The main features and results are presented in Table 3. Representative images of the technique are presented in Fig. 3. The mean follow-up ranged between 7 and 14.8 months.

**Table 3:** Summary of the characteristics and main results of clinical studies

Refs.	Study design	Tot. no. of patient	No. of test cases (bone lid)	Test cases - Biomaterial(s)	No. of control cases	Control cases - Biomaterial(s)	Mean follow-up duration (months)	Bone cutting instruments	Bone lid fixation	No. of implants (timing of insertion)	Assessment method(s)	Main findings
Johansson et al. [42]	RCT	24	10	None	19	Collagen membrane (CM) without bone graft [9] Autogenous bone graft (ABG) without membrane [10]	7	Piezosurgery	None Resorbable sutures (when further stability was searched)	Simultaneous implant placement (*n* = 101, comprising test and controls group)	Radiographic assessment: CBCT Clinical assessment Micro-CT of retrieved experimental implants Histological analysis (one retrieved experimental implant)	Clinical assessments: all lateral sinus walls ossified in ABG group, one and 2 lateral sinus walls not completely ossified in BW and CM groups, respectively Radiographic assessment: mean residual bone height in groups bone lid, CM, and ABG was 4.3 mm, 3.5 mm, and 4.3 mm, respectively, with no statistical difference found between these groups. No statistically significant correlations between sinus width (apicobuccal, *P* = 0.769; apicolingual, *P* = 0.532) and intra-sinus bone levels. Statistical difference between the apicobuccal distance and the apicolingual distance of the same implant. Mean apicobuccal distance/apicolingual distance was 0.6 mm/1.2 mm (bone lid group), 0.5 mm/0.8 mm (CM group), and 0.6 mm/0.8 mm (ABG group) (P = 0.003) Micro-CT: no statistical differences in %BIC between the groups (93.5% bone lid, 92.0% CM, and 93.5% ABG) Complications: one implant failure in the CM group
Sohn et al. [32]	Case–control study	10	5	None	5	Non-resorbable membrane	14.8	Piezosurgery	Fibrin adhesive None (if bone thickness > 1 mm)	Simultaneous implant placement (*n* = 21, comprising test and control group)	- Clinical assessments; Radiographic assessments (CT and plain radiograms) Histological analysis	Clinical assessments: no notable differences between test and control group in bone regeneration, but bone lid was more cost-effective and time-efficient as compared to non-resorbable membrane to seal the lateral wall of the sinus Radiographic assessments: All implants protruded a minimum of 4 mm into the sinus cavity; new bone formation behind original sinus floor Histological analysis: new bone formation in all cases Implant-related outcomes: all implants stable at the follow-up; implant survival rate 100% at follow-up Complications: 1 perforation (sealed with resorbable membrane and fibrin adhesive)

*RCT* randomized clinical trial

In the RCT [42], in all groups implant placement was performed simultaneously with the sinus floor augmentation using a two-stage protocol. No bone graft was placed after the elevation of the sinus membrane in the test group (bone lid), as well as in one of the control groups, in which the lateral window was closed with a resorbable membrane; while in a third group autogenous bone was used and the lateral access to the sinus was left open. Only in the latter, the lateral sinus wall was completely ossified, as confirmed by clinical observation at the second stage surgery.

In the case–control study [32], nongrafted sinus lift was performed in both groups and the replacement of the autogenous lateral bony window for the closure of the antrostomy was compared with a resorbable membrane. No significant difference was found in terms of bone regeneration between the two groups.

In all cases, the bony lid was repositioned without any fixation method, if adequate stability could be achieved. When further stabilization was required, the repositioned bony windows were fixed with resorbable sutures [42], while in Sohn et al. [32] fibrin adhesive was used when the bone lid was thinner than 1 mm.

In both studies, the cutting technique for the fashioning of the bone lid was the piezosurgery with a saw-shaped insert [32, 42]. Overall, only one membrane perforation was reported [32], and it was successfully managed with a resorbable membrane and fibrin adhesive.

In Johansson et al. [42], an additional implant was positioned in all patients and, after 7 months of healing, it was retrieved with the surrounding bone using a trephine bur. The samples were subjected to both micro-computed tomography (micro-CT) and subsequent histological analysis. Micro-CT data revealed no difference among the three investigated techniques in new intra-sinus bone formation. Furthermore, there were no statistical differences in bone-to-implant contact (BIC) between the groups, with mean values ranging between 92% and 93.5%. In Sohn et al. [32], similar new bone formation in the elevated sinus was detected in both test (i.e., bone lid) and control (i.e., non-resorbable membrane) patients after a mean healing time of 6 months from implant positioning and simultaneous sinus lifting. In all patients elevated sinus membrane was maintained tended by implants protruding within the sinus for at least 4 mm, as no bone substitute was utilized. It has to be noted that in this work a different bone sample retrieval method has been adopted. Indeed, bone biopsies were collected at the lateral access windows using a trephine bur during the second stage surgery after 4–8 months after surgery [32]. No differences in new bone formation were found between the two groups also at CT evaluation performed before the uncovering procedure.

In the RCT only one implant out of 101 was lost by the time of the second stage surgery after 7 months of healing [42], while in Sohn et al. [32] a survival rate of 100% was reported at 6 to 12 months from loading.

### Quality assessment and risk-of-bias analysis

For the selected animal studies, compliance with the updated ARRIVE guidelines [38] was evaluated for all the 21 items and provided in Table 4. In two studies all information were fully reported, except for the generalizability [29, 31]. Besides generalizability, in all the remaining animal studies [17–19], no information was reported about inclusion and exclusion criteria, blinding, housing and husbandry, animal care and monitoring, as well as protocol registration. All these three studies also presented criticisms regarding sample size calculation, randomization, experimental procedures and interpretation of data.

**Table 4:**
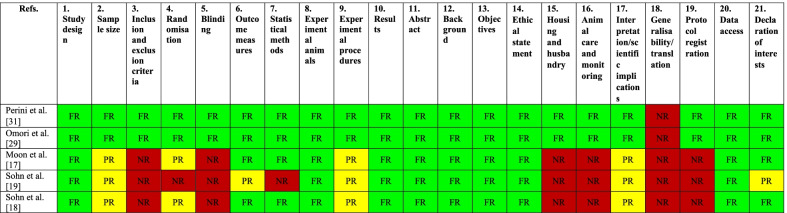
Quality assessment of the included in vivo animal studies using ARRIVE 2.0

*FR* fully reported (green), *PR* partially reported (yellow), *NR* not reported (red)

The risk-of-bias assessment of the included animal studies according to the SYRCLE tool [39] is presented in Table 5. In line with the quality assessment, only two animal studies presented low risk of bias [29, 31].

**Table 5:** Risk-of-bias assessment of the included in vivo animal studies using SYRCLE tool

Ref	1. Allocation sequence generation	2. Baseline characteristics	3. Allocation concealment	4. Random housing	5. Blinding of care giver/investigator	6. Random outcome assessment	7. Blinding of outcome assessor	8. Incomplete outcome data addressed	9. Free from selective outcome reporting	10. Free from other sources of bias
Perini et al. [31]	Yes	Yes	Yes	Unclear	Yes	Yes	Unclear	Yes	Yes	Yes
Omori et al. [29]	Yes	Yes	Yes	Unclear	Yes	Yes	Yes	Yes	Yes	Yes
Moon et al. [17]	No	Yes	No	Unclear	Unclear	Unclear	Unclear	No	Yes	Unclear
Sohn et al. [19]	No	Yes	No	No	No	No	No	No	Unclear	Unclear
Sohn et al. [18]	No	Yes	No	Unclear	Unclear	Unclear	Unclear	No	Yes	Unclear

The overall risk of bias of the RCT [42] resulted to be “some concerns” as reported in Table 6. Finally, the risk-of-bias assessment of the case–control study of Sohn et al. [32] is illustrated in Table 7, with 4 items judged as at “low risk” and 3 at “moderate risk”.

**Table 6:** Risk-of-bias assessment of the included RCT using RoB 2 tool

Refs.	1. Randomization process	2. Deviation from intended intervention	3. Missing outcome data	4. Measurement of the outcome	5. Selection of the reported outcome	6. Overall bias
Johansson et al. [42]	Low	Some concerns	Low	Some concerns	Low	Some concerns

**Table 7:** Risk-of-bias assessment of the included observational study using ROBINS-I tool

Refs.	1. Bias due to confounding	2. Bias in selection of participants into the study	3. Bias in classification of interventions	4. Bias due to deviations from intended interventions	5. Bias due to missing data	6. Bias in measurement of outcomes	7. Bias in selection of the reported result
Sohn et al. [32]	Moderate risk	Low risk	Low risk	Low risk	Moderate risk	Moderate risk	Low risk

### Meta-analysis

**Fig. 4:**
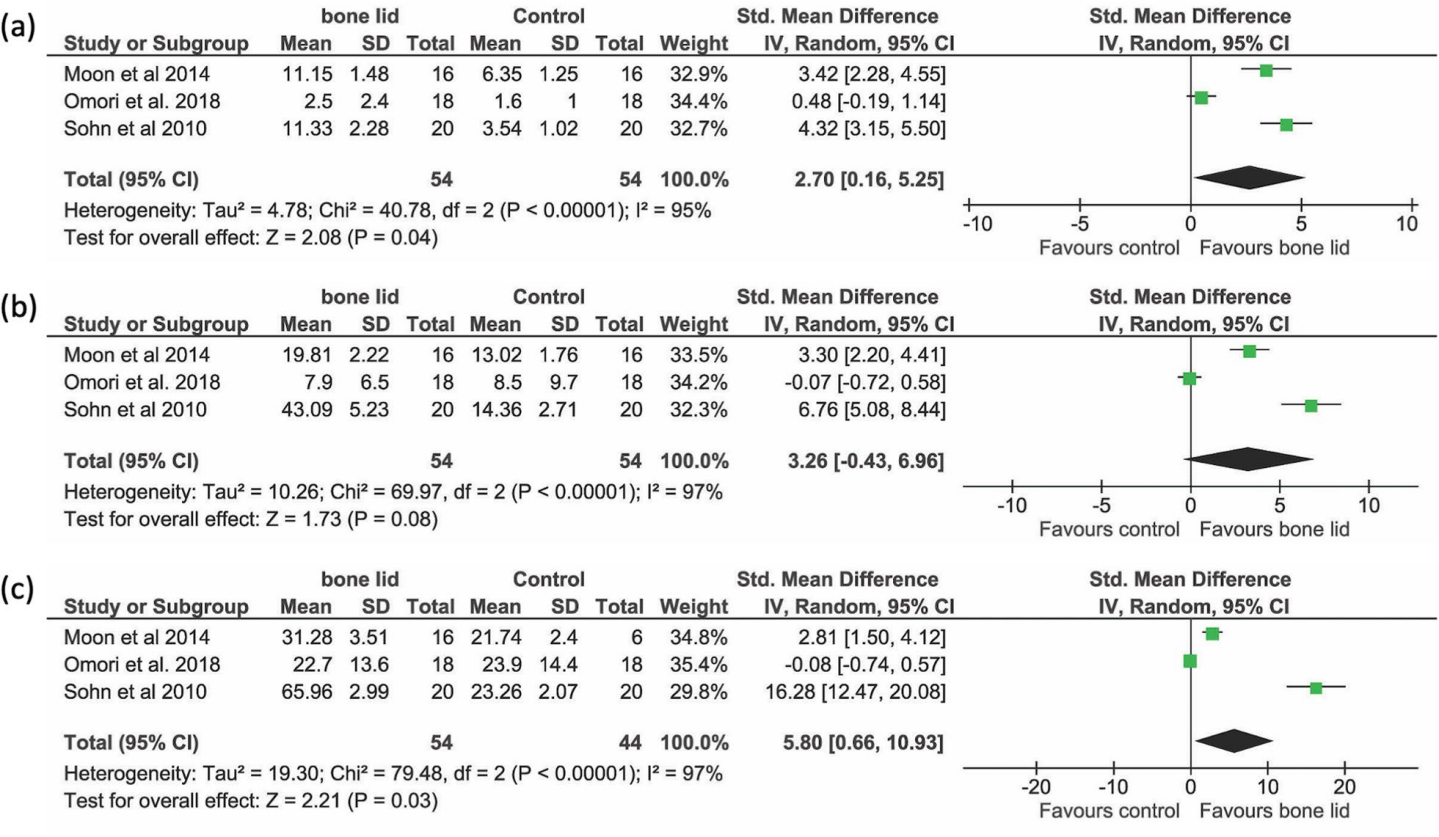
Forest plots from random‐effects meta‐analyses of the included animal studies on sinusal new bone formation after (**a**) two, (**b**) four, and (**c**) eight weeks of healing

A meta-analysis was done for the outcome “percentage of new bone formation”, by estimating the combined effect of three animal studies [17, 18, 29], after 2, 4, and 8 weeks (Fig. 4a–c). Since the heterogeneity among studies was significant, a random-effects model was used. There was a significantly higher new bone formation in the bone lid group after 2 weeks (*P* = 0.04) and 8 weeks (*P* = 0.03), while at 4 weeks the difference in favor of bone lid group did not achieve significance (*P* = 0.08).

## Discussion

This systematic review aimed to investigate the effect of the repositioned bone lid on bone augmentation in lateral sinus lift in animal and clinical studies.

Overall, pre-clinical studies confirmed the successful osseointegration of the repositioned bone lid after lateral sinus augmentation, which seems to promote new bone formation along the inner surface of the bone lid and support the *restitutio ad integrum* of the anterior maxillary wall. No bone lid dislocation was reported, except for one case, where the bone lid was lost, apparently during the histological processing [31]. The use of the bone lid was associated with a better healing, with reduced soft tissue ingrowth and enhanced new bone formation compared to controls, as confirmed by the meta-analysis conducted on three included studies in rabbits [17, 18, 29].

When the same biomaterial was grafted on both test and control side, differing only for the use of the bone lid or a resorbable membrane, higher and faster bone regeneration was generally observed on the bone lid side, as well as a reduced amount of soft tissue ingrowth into the elevated space [17, 29, 31]. The faster bone remodeling in presence of the bone lid was further confirmed by the significantly higher resorption of the β-TCP in the test side as compared to the contralateral control side after 8 weeks of healing [17]. In two studies in rabbits, the presence or the absence of the repositioned bone lid was not the only investigated variable, therefore it is not possible to quantify its contribution to the amount of newly formed bone [18, 19].

Rabbits were used in 4 out of the 5 (80%) included animal studies [17–19, 29]. This model can simulate with fair accuracy maxillary sinus augmentation in humans. However, functional loading of implants can be simulated only in larger animals [51].

None of the included pre-clinical studies have investigated bone remodeling by means of ex vivo and in vivo micro-CT [17–19, 29, 31]. In small animals, such as rodents and rabbits, multiple in vivo micro-CT scans can be obtained for a longitudinal examination of bone healing [52–54]. Indeed, this non-invasive high-resolution technique could have been of help in providing spatiotemporal information on the dynamic process of bone regeneration. Similarly, none of the included animal studies reported on the use of in vivo time-lapse microscopy to understand the dynamics of bone healing overtime [55].

No complications related to the bone lid were reported in both included clinical studies [32, 42]. Favorable outcomes in terms of bone lid healing and reintegration were overall reported, as confirmed clinically by the continuity of the lateral maxillary wall after 7 months of healing. Only in one case, the bone lid was partially ossified [42]. Histomorphometrical analyses revealed almost identical bone regeneration with or without the use of the bone lid technique [32, 42]. This seems to contradict the pre-clinical findings. However, contrary to animal studies, it is not possible in humans to thoroughly investigate the experimental site. Furthermore, it has to be noted that, despite histological analysis is reported in both clinical studies, a different biopsy technique was adopted. In Johansson et al. [42], bone samples were retrieved together with the osseointegrated experimental implants, while in the other study cylindrical bone biopsies were collected at the lateral access windows at the second stage surgery [32]. The latter approach on one side has the advantage to provide information on the healing of the bone lid, on the other side it does not allow the quantification of the peri-implant bone. In humans, core biopsies can also be harvested from the osteotomy for implant site preparation using trephine burs in case of delayed implant positioning [56].

In the present review, no membrane perforation was reported in all pre-clinical studies but one, where 3 perforations were observed in the control group and were all successfully managed with collagen membranes [29].

In clinical studies [32, 42], membrane perforation during bone lid fashioning or membrane elevation occurred in approximately 6.7% of the bone lid cases. This data agrees with the current literature on lateral sinus lift that reports a membrane perforation incidence ranging between 3.6% and 44% [57, 58].

The antrostomy can be prepared using various instruments. As piezosurgery was utilized in all clinical studies [32, 42] and in the majority of the pre-clinical ones [17–19], no meta-analysis could be performed to investigate the efficacy and the complications associated to different cutting tools. It is still controversial if the use of piezosurgery reduces the incidence of Schneiderian membrane perforations [58–62]. However, the piezosurgery handpiece with a saw-shaped insert, similarly to the micro-saw, allows the fashioning of thin osteotomies that are crucial for the fitting and stabilization of the bone lid at the original site [14, 24]. Indeed, when stabilization was obtained, no further fixation methods were used in both included clinical studies. This may decrease the length of the surgery, reduce the risk of mini-plate and screw exposure and avoid the need for a second surgery for their removal [14, 63, 64].

Clinical studies investigated survival rate of implants placed simultaneously with sinus lift, reporting high survival rates in both test and control sites. BIC could be determined only in one study, showing no statistically significant differences between the three groups [42]. Successful outcomes were reported for the bone lid technique combined with sinus membrane elevation tented by the concomitant implant insertion without bone substitutes. However, it has to be noticed that the studies had a follow-up shorter than one year, and implant-related outcomes were not considered as primary aims. Despite the report of a high implant survival rate in a recent systematic review and meta-analysis for both graftless and bone-grafted sinus lift groups, the absence of grafting material resulted in significantly lower bone density and vertical bone height gain [65]. It would be interesting to investigate in future clinical trials if using the bone lid technique could improve intra-sinus new bone formation and if it is correlated to a higher implant survival rate, both in case of simultaneous and delayed placement.

Limitations of this systematic review and meta-analysis are the limited number of included studies, the moderate-to-high risk of bias of several included studies and the lack of a uniform standard control treatment. Finally, concerns regarding the transferability of animal studies to humans have to be mentioned, due to the different sinus anatomy, the different assessment methods and the impossibility to assess implant survival rate especially in small animal models.

## Conclusions

Despite the limitations of the present work, it can be concluded that the repositioned bone lid combined with lateral maxillary sinus lift presented a low percentage of complications, in both pre-clinical and clinical studies. The meta-analysis conducted only for animal studies provided moderate evidence that the repositioned bone lid favored the formation of new bone to a higher extent as compared to resorbable membranes in animal studies. Despite being not possible to histologically investigate the whole regenerated sinus in humans, animal data should be confirmed in clinical trials, for instance by means of high-resolution 3D imaging. Overall, the repositioned bony windows were found to be well integrated and fixed to the lateral sinus wall. Clinical trials are needed to assess the long-term success of dental implants in combination with the investigated technique.

## Data Availability

Not applicable.

## Abbreviations

β-TCP: β-Tricalcium phosphate
BIC: Bone-to-implant contact
CBCT: Cone beam computed tomography
DBBM: Deproteinized bovine-derived bone mineral
RCT: Randomized clinical trial
