# Correction to: The bone lid technique in lateral sinus lift: a systematic review and meta-analysis

**DOI:** 10.1186/s40729-022-00444-0

**Published:** 2022-10-10

**Authors:** Lucia Schiavon, Alessandro Perini, Giulia Brunello, Giada Ferrante, Massimo Del Fabbro, Daniele Botticelli, Fouad Khoury, Stefano Sivolella

**Affiliations:** 1 Department of Neurosciences, Dentistry Section, University of Padova, Via Giustiniani 2, 35128 Padua, Italy; 2 Department of Oral Surgery, University Clinic of Düsseldorf, Moorenstrasse 5, 40225 Düsseldorf, Germany; 3 Department of Biomedical, Surgical and Dental Sciences, Università Degli Studi di Milano, Via Commenda 10, 20122 Milan, Italy; 4 Fondazione IRCCS Ca’ Granda Ospedale Maggiore Policlinico, Via Francesco Sforza, 35, 20122 Milan, Italy; 5 ARDEC Academy, Viale Pascoli 67, Rimini, Italy; 6 Department of Oral and Maxillofacial Surgery, University of Munster, Waldeyerstr. 30, 48149 Münster, Germany; 7 International Dental Implant Center, Private Clinic Schloss Schellenstein, Am Schellenstein 1, 59939 Olsberg, Germany

## Correction to: International Journal of Implant Dentistry (2022) 8:33 https://doi.org/10.1186/s40729-022-00433-3

Following publication of the original article [1], we have been notified that authors’ first and last names were mentioned incorrectly and they should be switched as per below:

- Lucia (first name) Schiavon (last name)
- Alessandro (first name) Perini (last name)
- Giulia (first name) Brunello (last name)
- Giada (first name) Ferrante (last name)
- Massimo (first name) Del Fabbro (last name)
- Daniele (first name) Botticelli (last name)
- Fouad (first name) Khoury (last name)
- Stefano (first name) Sivolella (last name)
